# An RPA-Based CRISPR/Cas12a Assay in Combination with a Lateral Flow Assay for the Rapid Detection of *Shigella flexneri* in Food Samples

**DOI:** 10.3390/foods13193200

**Published:** 2024-10-09

**Authors:** Jieru Xu, Tianxin Zhang, Xinrui Lv, Lei Shi, Weibin Bai, Lei Ye

**Affiliations:** 1 Institute of Food Safety and Nutrition, Jinan University, Guangzhou 510632, China; 2 Shandong Yuwang Ecological Food Industry Co., Ltd., Yucheng 251200, China

**Keywords:** *Shigella flexneri*, RPA, CRISPR/Cas12a, lateral flow detection, food safety

## Abstract

Among the pathogens that cause infectious diarrhea in China, *Shigella* is the most prominent. Shigellosis affects both adults and children, particularly those in developing nations, with nearly 190 million annual cases and a third resulting in fatalities. The recently emerged CRISPR/Cas system has also been increasingly applied for the detection of different biological targets. The lateral flow assay (LFA) has the advantages of short detection time, simple operation, high sensitivity, and low cost, and it provides an ideal platform for on-site detection. In this study, a recombinase polymerase amplification–CRISPR/Cas12a–LFA test for *Shigella flexneri* was constructed. The established method had good specificity and sensitivity, and the qualitative accuracy of 32 tested strains reached 100%. The detection limit of genomic DNA reached 8.3 copies/μL. With the advantages of high accuracy and portability, this diagnostic apparatus represents a novel method of identification and detection of *Shigella flexneri*, particularly in settings that lack complex laboratory infrastructure.

## 1. Introduction

*Escherichia coli* has long been the focus of intense scrutiny, with it serving as a pivotal marker of human health [1]. The ability to detect this bacterium rapidly and with great sensitivity is crucial to ensure food safety [2]. The Enterobacteriaceae family encompasses critical genera such as *Escherichia*, *Salmonella*, and *Shigella* [3]. Among the pathogens causing infectious diarrhea in China, *Shigella* stands at the forefront [4]. Shigellosis affects both adults and children, particularly those in developing nations [5], with nearly 190 million annual cases and a third resulting in fatalities [6]. *Shigella* can be categorized into four primary serotypes: *Shigella flexneri*, *Shigella sonnei*, *Shigella boydii*, and *Shigella dysenteriae*, each with varying incidence rates across different regions [7]. In developing countries, *S. flexneri* is characterized by its toxicity and epidemic propensity [8]. It typically manifests as watery or bloody diarrhea. It possesses a robust environmental resilience, thriving in common settings such as salads, bread, and ready-to-eat meals [9].

To forestall epidemics of *S. flexneri* and preserve food safety, the prompt and sensitive detection of this pathogen is imperative for rapid containment of outbreaks [10]. However, the current gold-standard detection methods for *S. flexneri* rely on sophisticated instrumentation and skilled technicians, limiting their application to centralized laboratories [11]. Thus, there is a dire need for simple, rapid, and affordable detection techniques to empower healthcare providers with accurate diagnostics for *S. flexneri* [12].

Clustered regularly interspaced short palindromic repeats (CRISPR) and associated proteins (Cas) under the specific guidance of single-guide RNA (sgRNA) leverage the trans-cleavage activity of Cas12a to non-specifically cleave single-stranded DNA [13]. The integration of CRISPR/Cas12a with isothermal amplification can realize a versatile platform for rapid and specific molecular detection [14], such as fluorescence-based assays and visually readable lateral flow assays [15]. Most notably, these methodologies can deliver sensitivity comparable to or surpassing real-time PCR without the need for a complex apparatus, rendering them highly suitable for point-of-care diagnostics [16].

With the evolution of detection technologies, isothermal amplification is a current focal point of research [17]. To detect *S. flexneri*, efforts have primarily converged on the amalgamation of CRISPR/Cas12a with loop-mediated isothermal amplification (LAMP) or CRISPR/Cas13a with recombinase polymerase amplification (RPA) [18]. Within the CRISPR system, both Cas12a and Cas13a perform trans-cleavage following cis-cleavage, but while Cas12a exerts effective trans-cleavage activity on ssDNA, Cas13a targets ssRNA [19]. Therefore, in our experiments, Cas12a is the protein of choice. Isothermal amplification by recombinant polymerase at 37 °C requires a short period (approximately 30 m). This is especially compelling [20]. Compared with other amplification methods such as LAMP and rolling circle amplification (RCA), RPA is devoid of the need for temperature control and intricate primer design, and it is supported by ready-to-use lyophilized reagent kits [21]. It is better suited for truly portable rapid nucleic acid testing [22]. Shi, Kang et al., 2022 successfully developed the LAMP-CRISPR/Cas12a method for detecting *Shigella flexneri*, demonstrating both high sensitivity and specificity. However, RPA offers greater convenience and cost-effectiveness compared to LAMP, primarily because it requires only a single pair of primers, whereas LAMP necessitates four to six primers. Moreover, RPA amplification and Cas12a detection are compatible at the same temperature. Consequently, the RPA–CRISPR/Cas12a method, which operates effectively at 37 °C with just one pair of primers, presents a streamlined approach in contrast to LAMP, which requires 65 °C and multiple primer sets. These characteristics make RPA–CRISPR/Cas12a particularly advantageous for field applications [23].

In this study, we synthesize a detection method for *S. flexneri* that boasts high specificity and sensitivity by uniting RPA, CRISPR/Cas12a, and LFAs. This method commences with isothermal amplification at 37 °C, followed by integration with CRISPR/cas12a reagents for cutting. This portable and simple instrument allows for the rapid, sensitive, and specific detection of *Shigella flexneri*. Even in resource-poor environments, such as developing countries, it will not cross-react with other common pathogens. Consequently, the RPA–CRISPR/Cas12a–LFA system is an expedient tool for the swift identification of *S. flexneri* pathogens in resource-constrained settings. It has great potential in the point-of-care testing of foodborne pathogens to prevent foodborne diseases and ensure food safety.

## 2. Materials and Methods

### 2.1. Bacterial Culture and Genomic DNA Extraction

*Shigella flexneri* (ATCC 12022) was initially revived in a nutrient broth, and then the single colony was isolated in MacConkey agar and cultured in lysogeny broth (LB). According to the manufacturer’s guidelines, gDNA was extracted from these cultivated strains using a bacterial genomic DNA extraction kit (Vazyme Biotech Co., Ltd., Nanjing, China). The concentration and purity of the extracted DNA were quantified using a NanoDrop(Thermo Fisher Scientific Shier Technology Co., Ltd., Shanghai, China). Tenfold dilutions of the DNA were prepared for further experiments. The extracted genomic DNA was stored at −20 °C for future use [24].

### 2.2. Design of Primers, sgRNA, and ssDNA Probes

In this study, the highly conserved hypothetical protein gene of *Shigella flexneri* was selected as the target for the RPA primers and crRNA [25]. The genes selected are conserved in the relevant serotypes, to cover multiple serotypes. The virulence gene ipaH was selected as the target of PCR detection. The RPA primers were designed using Primer Premier 5 and synthesized by Sangon Biotech (Shanghai, China). Prior to their use, their sequences were subjected to BLAST analysis via GenBank to verify their specificity. Based on the specific recognition of the TTTN sequence (PAM), crRNA was designed and synthesized by Shanghai Diying Biotechnology Co., Ltd. (Shanghai, China). The ssDNA FAM-Biotin (FB) and FAM-BHQ (FQ) probes were designed to detect fluorescence and visualize the LFA (Table 1).

### 2.3. PCR/qPCR/RPA Amplification Reaction

The PCR mix included 10 µL of EasyTaqPCR Super Mix, 0.4 µM primers, 2 µL of gDNA template, and up to 20 µL of nuclease-free water. The amplification began with 5 min of pre-denaturation at 94 °C, followed by 30 cycles of 30 s at 94 °C, 58 °C, and 72 °C, and ending with a 7 min extension at 72 °C. The sample was run on 1.5% agarose gel for final product analysis. The qPCR mix contained 0.2 μM primers, 10 μL of premix, 2 μL of gDNA template, and up to 20 μL of ddH_2_O. It was run on an ABI QuantStudio6 Q6 system to detect fluorescence signals. The amplification involved an initial denaturation at 95 °C for 30 s, followed by 45 cycles of 5 s at 95 °C, 30 s at 60 °C, and 35 s at 60 °C. RPA detection used an isothermal amplification kit (Nanjing Wobo Biotechnology Co., Ltd., Nanjing, China). The reaction mixture, containing 2 μL of DNA, 2 μL of SF–RPA–F1/R1 primers (10 μM), 2.5 μL of magnesium acetate, 29.4 μL of RPA buffer, and 12.1 μL of ddH_2_O, was incubated at 37 °C [26].

### 2.4. Visualization Platform for RPA–CRISPR/Cas12a–LFA Detection

The RPA–CRISPR/Cas12a–LFA reaction included 2 μL of a 10× reaction buffer, 200 nM of LbaCas12a protein, 100 nM of crRNA, 500 nM of a ssDNA-FQ reporter molecule, and nuclease-free water. The RPA product was added to the CRISPR–Cas12a complex and centrifuged for mixing [27]. It was then incubated at 37 °C for 30 min in the DHelix-Q5 instrument (Guangzhou Double Helix Gene Technology Co., Ltd., Guangzhou, China). As the ssDNA was cleaved, the fluorescence was recorded every 30 s using the DHelix-Q5 to monitor the reaction in real time. We devised a visualization platform capable of detecting *Shigella flexneri*. This LFA-based detection employed the same reagents as the fluorescence-based detection but substituted the FQ reporter with an FB reporter. The reaction proceeded in a 1.5 mL tube containing Cas12a and crRNA [28]. After an initial 10 min incubation, the reaction mixture that included the FB reporter, Cas buffer, and DNA template was incubated at 37 °C for 30 min. Subsequently, a lateral flow strip was introduced to the tube, and results were read within 10 min. Visual observation of the control line and test line on the lateral flow strip was used for qualitative evaluation. The control band was positioned near the sample pad, while the test band appeared at the top of the strip, distanced from the sample pad. The emergence of a red test band indicated a positive result, and all other outcomes were considered negative. The blue part is the name of the manufacturer of the test strip [29].

### 2.5. Specificity and Sensitivity Assessment of the RPA–CRISPR/Cas12a–LFA System

To validate the specificity of RPA–CRISPR/Cas12a–LFA, we used *Shigella flexneri* as a positive control contrasted against 14 non-*Shigella flexneri* species that included strains of *Escherichia coli* and various *Pseudomonas* and *Vibrio* as negative controls, with ddH_2_O providing a no-template baseline (Table 2). These specimens were methodically compared with the established qPCR benchmark. To ascertain the sensitivity of the RPA–CRISPR system, we conducted a tenfold serial dilution of *Shigella flexneri* genomic DNA (8.3 × 10^6^ to 8.3 copies/µL) and evaluated it against the gold-standard qPCR.

### 2.6. Application of the Shigella flexneri RPA–CRISPR/Cas12aLFA Detection Method to Artificially Contaminated Samples

Sterile milk was validated to be free of *Shigella flexneri* via high-pressure pasteurization. Prior to the experiment, the presence of *Shigella flexneri* in the samples was confirmed using qPCR. A 25 mL sample was placed in a sterile sampling bag, and 225 mL of sterile PBS was added. The sample was manually homogenized using a pestle until a uniform mixture was achieved. To evaluate the detection limit of the RPA–CRISPR/Cas12a–LFA for artificially contaminated milk, 1 mL of *Shigella flexneri* at various concentrations was mixed with 9 mL of the homogenized sample to prepare *Shigella flexneri* samples ranging from 5.6 × 10^7^ to 5.6 × 10^1^ CFU/mL. Next, 1 mL aliquots were taken from each gradient, and DNA extraction was performed as described in Section 2.1. The extracted DNA was then used as the template for the RPA–CRISPR/Cas12a–LFA assay. Each food sample underwent two rounds of testing with the RPA–CRISPR/Cas12a–LFA, and the experiment was repeated three times.

### 2.7. Validation of the RPA–CRISPR/Cas12a–LFA System with Actual Samples

To appraise the feasibility of the RPA–CRISPR/Cas12a–LFA methodology, we obtained 30 food samples from the local market (Guangzhou, Guangdong Province, China), including 10 bananas, 10 different brands of milk, and 10 salad samples. To each 25 g food sample in a sterile sampling bag, we added 225 mL of sterile LB broth, manually homogenized the mixture to ensure uniformity, and then incubated it at 37 °C. Genomic DNA was subsequently extracted from 1 mL of the homogenized solution and utilized as a template for both the RPA–CRISPR/Cas12a assay and qPCR analysis.

### 2.8. Data Analyses

The qPCR Ct values were generated using the CFX96 Touch^TM^ Real-Time PCR Detection System and are reported as means ± standard deviations. For RPA and CRISPR experimental data, statistical analyses were conducted with SPSS Statistics 23.0, while graphs were produced using OriginPro, version 8.5. All experiments were performed in triplicate to ensure reproducibility, and error bars represent the standard deviations derived from at least three independent experiments. Each sample was measured in triplicate to maintain consistency and reliability of the results.

## 3. Results and Discussion

### 3.1. Working Principle of the RPA–CRISPR/Cas12a–LFA Detection System

**Figure 1:**
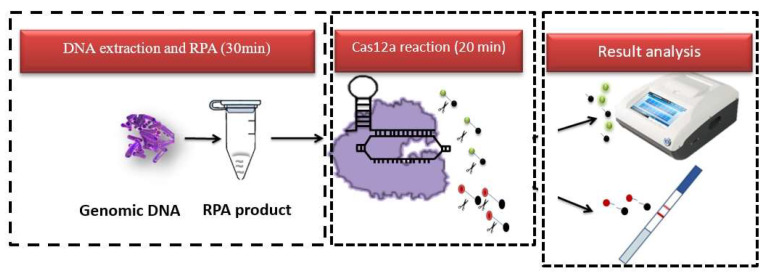
A schematic representation of the RPA–CRISPR/Cas12a–LFA principle. The green ball and the red ball represent two kinds of probes used by fluorescence and test paper respectively, which emit fluorescence after being cut.

To enhance the rapid, precise, and sensitive detection of *Shigella flexneri*, we developed an RPA–CRISPR/Cas12a–LFA detection system. This system integrated the LbCas12a protein, tailored CRISPR/crRNAs, and targeted nucleic double-stranded DNA. A single-stranded DNA fluorescent probe served as a fluorescent reporter within the ternary complex. Additionally, for direct visual assessment, the FB reporter gene was used for the lateral flow strip analysis, enhancing both sensitivity and specificity (Figure 1). This lateral flow system offers a rapid, cost-effective analysis and operates with simplicity, obviating the need for specialized technical skills. After applying the Cas12a reaction solution to the sample application area at the bottom of the lateral flow strip, the absence of target DNA caused the anti-FAM capture antibody to bind to the control line, resulting in a visible band only at the control line [30]. Therefore, the color band only appeared on the control band. In contrast, when the target was present, the DNA and FB-reporter gene were cut by the activated trans-cutting activity. The dissociated FAM then flowed through the control zone and was captured by anti-rabbit antibodies. Therefore, the color band only appeared on the test strip. In the case of low abundance, the target DNA and some unbroken FB-reporter molecules would stick to the control band, and the color bands would exist in both the control band and the test band.

### 3.2. Optimization of the RPA Reaction for the RPA–CRISPR/Cas12a–LFA System

Nucleic acid amplification is pivotal for the efficacy of detection methodologies that use the CRISPR/Cas system. Distinct from PCR and LAMP, RPA facilitates the rapid amplification of nucleic acids at a constant temperature, and this substantially reduces the dependency on sophisticated instrumentation and precise thermal cycling. Consequently, RPA is particularly advantageous in non-laboratory and resource-constrained environments. To identify the most effective primer pair, we synthesized three forward and reverse primers, as shown in Table 1. Each primer combination was assessed using RPA at 37 °C for 30 m. Subsequently, one of the three additional primer sets exhibited the most significant fluorescence in the CRISPR–Cas12a assay. The optimal primer pair was selected for subsequent detection experiments targeting *Shigella flexneri*. During the subsequent process of specificity determination, the specificity of the first pair of primers was better than that of the other two pairs of primers. Therefore, the first pair of primers was selected for the subsequent experiment.

**Figure 2:**
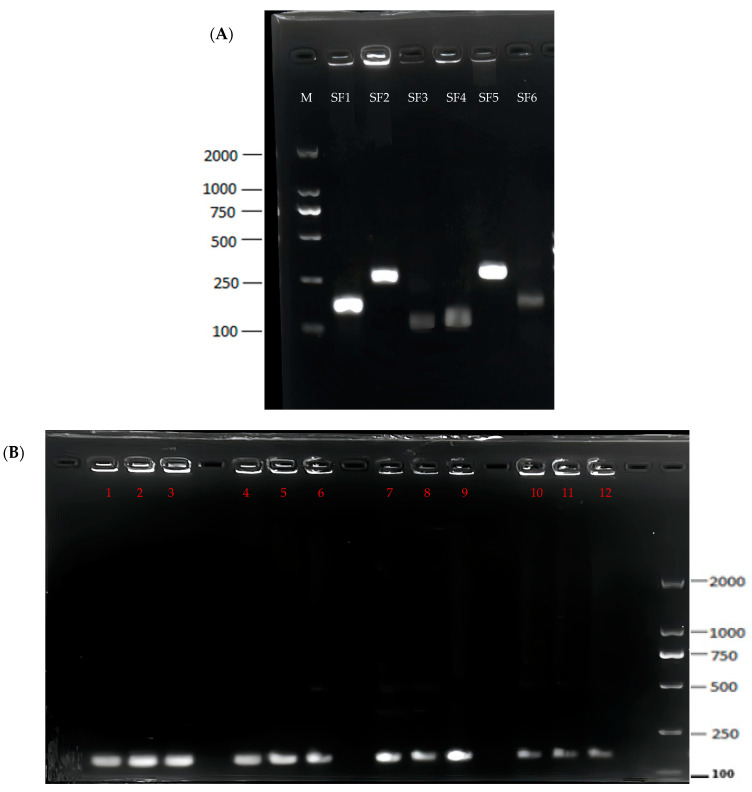
Screening of RPA primers and optimization of the RPA system. (**A**) The amplified products of each RPA primer combination were visualized under 1.5% agarose gel electrophoresis. M: Maker, Line 1: F1R1, Line 2: F1R2, Line 3: F1R3, Line 4: F2R1, Line 5: F2R2, and Line 6: F2R3. (**B**) Optimization of the RPA system. 1: 35 °C, 25 min; 2: 35 °C, 35 min; 3: 35 °C, 45 min; 4: 36 °C, 25 min; 5: 36 °C, 35 min; 6: 36 °C, 45 min; 7: 37 °C, 25 min; 8: 37 °C, 35 min; 9: 37 °C, 45 min; 10: 38 °C, 25 min; 11: 38 °C, 35 min; and 12: 38 °C, 45 min.

We also sought to refine the amplification conditions of RPA to maximize efficiency within the minimal temporal scope. Our investigations indicated that temperatures of 35 °C and 38 °C supported robust amplification across durations of 25, 35, and 45 m. Optimizing the reaction parameters to 36 °C for 25 m not only reduced the reaction duration but also augmented the signal intensity while preserving the stability of the RPA reaction (Figure 2B). For enhanced field diagnostic development and applications, a temperature and time setting of 36 °C for 25 m was selected as ideal.

### 3.3. Sensitivity and Specificity of the RPA Reaction

**Figure 3:**
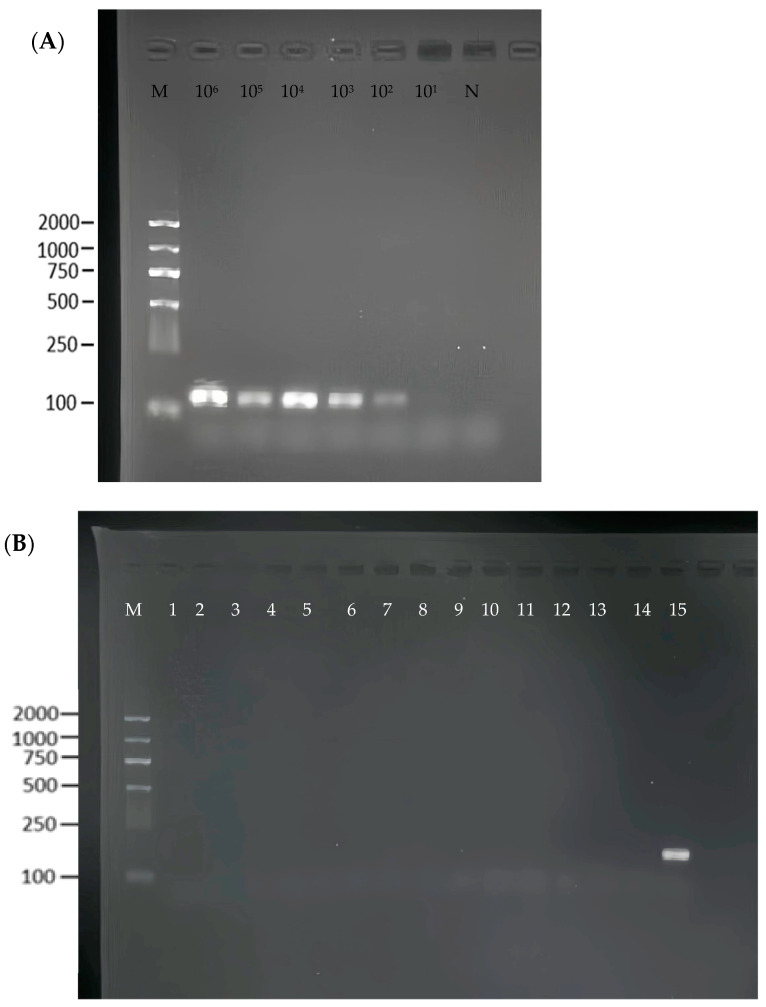
Specificity and sensitivity of the RPA reaction. (**A**) 1–6: serial dilution bacterial suspension, N: negative control, M: Maker. (**B**) 1–14: non-*Shigella flexneri*, 15: *Shigella flexneri*.

To assess the sensitivity of the RPA method, a series of tenfold dilutions of *S. flexneri* recombinant plasmids (10^6^–10^1^ copies/μL) were used as templates. As shown in Figure 3A, the limit of detection of the RPA was 10^2^ copies/μL. We used standard *Shigella flexneri* and 14 strains of non-*Shigella flexneri* for the specificity determination and found that the RPA reaction had good specificity (Figure 3B).

### 3.4. Feasibility of crRNA-Guided Cis- and Trans-Cleavage by Cas12a

The complementarity between crRNA and the DNA target within the RPA-amplified region is crucial for activating Cas12a’s trans-cleavage activity. This activation occurs only when both Cas12a/crRNA and the target DNA are present, facilitating the cleavage of the ssDNA-FQ fluorescent probe and resulting in significant fluorescence that is detectable using fluorescence imaging devices. To maximize the signal readout during Cas12a-mediated trans-cleavage, we initially assessed the detection system’s essential components. The detection system results demonstrated that essential components include RPA amplification products, fluorescent probes, Cas12a, and crRNA. Omission of any of these elements would lead to the system’s inability to detect positive signals (Figure S1). This outcome provided compelling evidence in support of the RPA–CRISPR/Cas12a principle. Subsequent optimization focused on the optimal ratio of Cas12a to crRNA, the quantity of the RPA amplification product, and the dosage of the fluorescent probe.

### 3.5. RPA–CRISPR/Cas12a Assay Optimization

The proportion of Cas12a to crRNA significantly influences the utilization rate of Cas12a. An optimal amount ensures high fluorescence generation efficiency, thus enhancing detection capability while being cost-effective. The experiment was repeated more than three times. The optimal fluorescence signal was achieved with a 2:1 molar ratio of Cas protein to crRNA (Figure S2A), leading to the use of 200 nM Cas12a and 100 nM crRNA in subsequent experiments.

An insufficient amount of RPA amplification product would fail to generate adequate reportable signals, while an excessive amount of amplification product would introduce unnecessary components into the system (Figure S2B). The fluorescence signals plateaued when the amplification product volume reached 5 µL and declined at 6 µL. The results of Figure S2B show that increasing the amount of RPA product does not lead to a linear increase in fluorescence signals, which may be caused by several factors. Firstly, the fluorescence signal of the CRISPR/Cas12a system will reach saturation under certain conditions, and beyond this limit, the fluorescence signal is no longer directly proportional to RPA products. In addition, the concentration of other components in the reaction system, such as Cas12a protein or crRNA, may also limit the further increase of signal. The experiment was repeated more than three times. Thus, 5 µL was established as the optimal volume of RPA product.

A precise quantity of the fluorescent probe maximizes the fluorescence signal, thereby enhancing the system’s detection capacity. The experiment was repeated more than three times. Excessive probe volumes, however, increase costs (Figure S2C). Peak fluorescence signals were achieved with 5 µL of the probe, and this volume was selected to balance signal maximization and cost efficiency.

### 3.6. Sensitivity and Specificity Evaluation of the RPA–CRISPR/Cas12a–LFA Platform

Using the non-specific cleavage capabilities of the Cas12a protein, a novel detection system for *Shigella flexneri* was engineered. This system integrated RPA and CRISPR/Cas12a technologies to transform nucleic acid sequence information into visually interpretable signals. Following the refinement of the RPA reaction and the CRISPR/Cas12a cleavage response, we established the CRISPR/Cas12a-RPA methodology, whose specificity and sensitivity were rigorously assessed.

**Figure 4:**
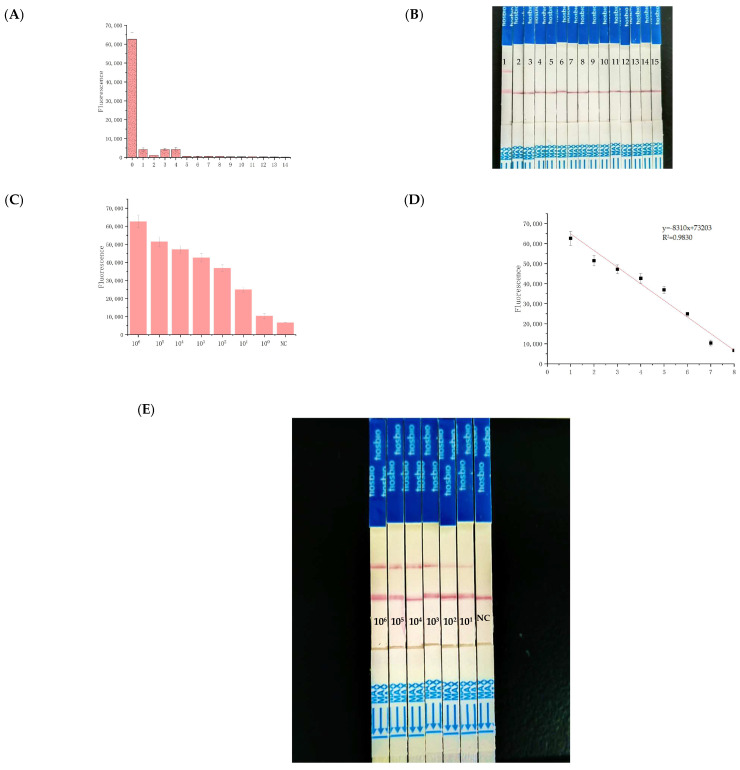
Evaluation of the specificity and sensitivity of the RPA–CRISPR/Cas12a–LFA system: (**A**) Specificity test results. (**B**) 1: *Shigella flexneri*, 2–15: non-*Shigella flexneri*. (**C**) sensitivity evaluation results of the bacterial suspension template. (**D**) linear relationship between the fluorescence intensity and the concentration of *Shigella flexneri* over the range of 8.3 × 10^6^ to 8.3 × 10^1^ copies/µL. (**E**) naked-eye detection of CRISPR/Cas12a-LFA. NC: Negative Control.

A range of pathogens was tested, including five Escherichia coli strains, three Vibrio species, two Pseudomonas aeruginosa strains, and singular instances of *Staphylococcus aureus*, *Cronobacter sakazakii*, *Listeria monocytogenes*, and *Bacillus cereus*. The results confirmed the absence of cross-reactivity, except for the *Shigella flexneri* genome, thereby demonstrating the high specificity of the RPA and crRNA primers used within the CRISPR/Cas12a framework (Figure 4A). Supplementary Data provided via qPCR illustrated that all non-*S. flexneri* organisms returned a negative fluorescence result. We used standard *Shigella flexneri* and 14 strains of non-*Shigella flexneri* for the specificity determination and found that the RPA–CRISPR/Cas12a–LFA reaction had good specificity (Figure 4B).

To establish the method’s sensitivity, *Shigella flexneri* genomic DNA was serially diluted tenfold (ranging from 8.3 × 10^6^ to 8.3 copies/µL) and tested against standard qPCR and RPA (Figure 3A). The detection threshold for qPCR (Figure S3) was identified at 8.3 × 10^2^ copies/µL (Figure S4), whereas for the RPA–CRISPR/Cas12a–LFA system, a remarkably low limit of 8.3 copies/µL was observed (Figure 4C). Using LFA biosensors, the detection limit for *Shigella flexneri* was approximated at 83 copies/µL (Figure 4E). In summary, *Shigella flexneri* can be detected with high sensitivity using the CRISPR/Cas12a system. Compared to most previously reported CRISPR/Cas-based diagnostic methods, it is noted for its superior sensitivity and strong quantitative detection capabilities, making it a compelling option for advanced diagnostic applications (Table S1).

### 3.7. Sensitivity of the RPA–CRISPR/Cas12a–LFA Method in Artificially Contaminated Samples

**Figure 5:**
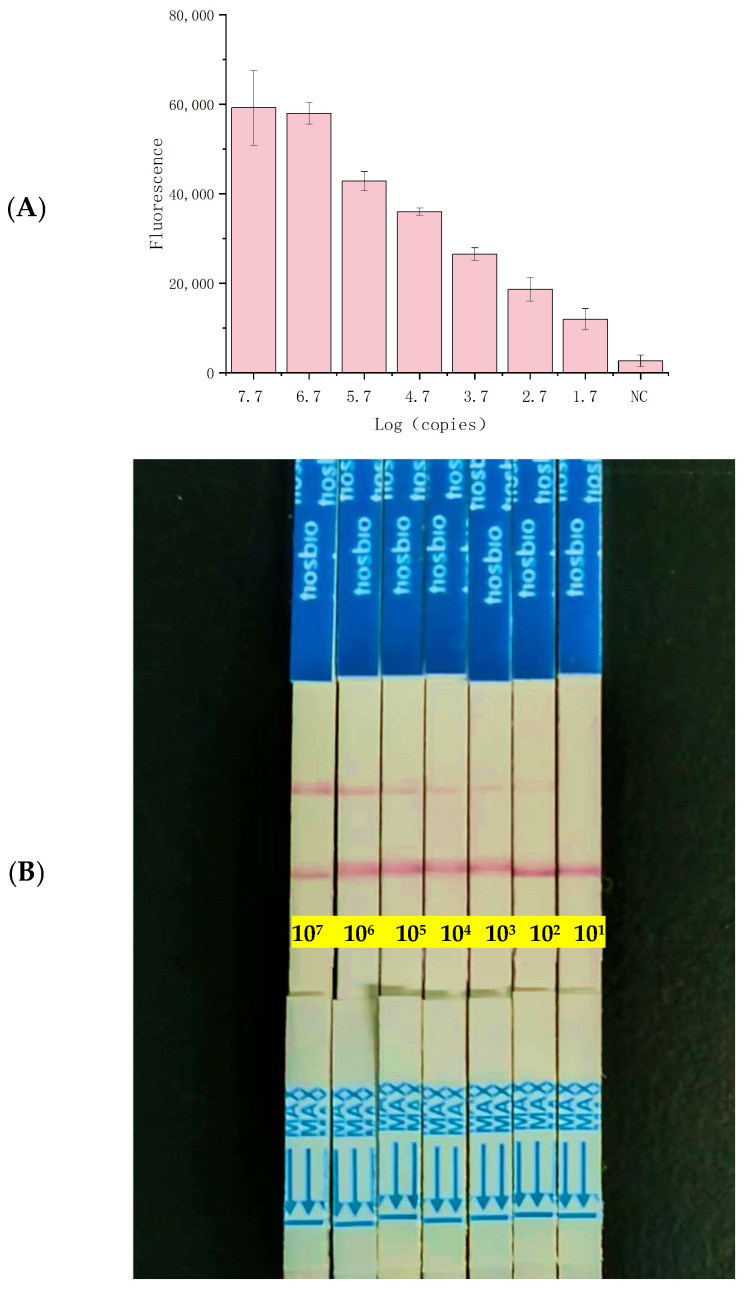
Limit of detection (LOD) of the RPA–CRISPR/Cas12a–LFA system for *Shigella flexneri* in milk. (**A**) Fluorescence curve. (**B**) sensitivity of the RPA–CRISPR/Cas12a–LFA assay for *Shigella flexneri* detection. NC: Negative Control.

To evaluate the applicability of the CRISPR/Cas12a detector for food safety surveillance, artificially contaminated milk samples (ranging from 5.6 × 10^7^ to 5.6 × 10^1^ CFU/mL) were analyzed using the RPA–CRISPR/Cas12a detector system (Figure 5A). Even at a concentration of 5.6 × 10^1^ CFU/mL in milk samples, the RPA–CRISPR method generated fluorescence signals significantly distinct from the control group. In the LFA biosensor trials, samples containing 5.6 × 10^2^ CFU/mL exhibited faint test lines, indicating that detection was feasible at levels as low as 560 CFU/mL (Figure 5B). These findings underscore the rapidity and sensitivity of the RPA–CRISPR/Cas12a–LFA technique.

### 3.8. Evaluating the Consistency between RPA–CRISPR/Cas12a–LFA and qPCR in Actual Samples

To further explore the practicality of this method, 32 real-world samples (Table S2), including salads, bananas, and eggs, were processed to extract the genomic DNA of *Shigella flexneri* isolates. These samples underwent RPA at 35 °C for 30 m, followed by mixing with preloaded CRISPR/Cas12a reagent at 37 °C for 20 m. Additionally, the extracted genomic DNA of these *S. flexneri* isolates was verified using the gold-standard qPCR method. As shown in Figure S5 all *S. flexneri* field isolates were positively identified by our CRISPR/Cas12a-RPA–LFA system as indicated by the test strip. This was corroborated by positive qPCR findings for all isolates (Table S3). This comprehensive validation demonstrated the CRISPR/Cas12a-RPA–LFA system’s efficacy in field diagnostics, highlighting its potential to streamline pathogen detection in complex sample matrices.

## 4. Conclusions

We developed a rapid and sensitive LFA system based on RPA and CRISPR/Cas12a that was capable of naked-eye detection of *Shigella flexneri*. The results indicated that the novel assay was suitable for use with a fluorescence reader. The detection limit of the RPA–CRISPR/Cas12a–LFA assay for *Shigella flexneri* was 83 copies/μL at 36 °C for 25 min, as demonstrated by naked-eye detection under an ultraviolet transilluminator. The RPA–CRISPR–Cas12a method showed high sensitivity in detecting *S. flexneri* in both lab and food samples, demonstrating its practical utility. This method offers enhanced sensitivity and specificity compared to the LAMP–CRISPR approach. Although LAMP–CRISPR achieves a sensitivity of 4 × 10^0^ copies/μL, it requires a higher operating temperature and the use of more than three pairs of primers, making it less straightforward than RPA. Additionally, this study combines RPA–CRISPR/Cas12a with lateral flow test strips, an affordable real-time fluorescence detection tool. This method is particularly suitable for point-of-care testing (POCT) in resource-limited settings. Therefore, the primary advantages of the RPA–CRISPR/Cas12a–LFA assay are as follows: (1) the operational processes are straightforward, and no sophisticated equipment is required; (2) This study is the first to use RPA–CRISPR/Cas12a combined with lateral flow analysis (LFA) to detect *Shigella flexneri* in food samples; (3) the sensitivity of the detection process is high, and the signal can be visually interpreted. This makes this method accurate, portable, and highly applicable in the context of food safety, particularly in a field setting, without the necessity of highly technical expertise or expensive laboratory equipment.

## Figures and Tables

**Table 1:** Sequences of the RPA primers and CRISPR guide RNA (CrRNA).

Name	Sequence
SF1-F	GCCACGACTATGCTGTAACTTTCCCGGATG
SF1-R	CTTACCGCCAATCTCTTCGGAGGCAGCTGA
SF2-F	CGATAATGATACCGGCGCTCTGCTCTCCCTG
SF2-R	CTTCCAGACCATGCTCGCAGAGAAACTTCAG
SF3-F	CTGCATGGCTGGAAAAACTCAGTGCCTCTG
SF3-R	GTTCTGACTTTATCCCGGGCAATGTCCTCC
crRNA	GAAUUUCUACUGUUGUAGAUUGGUCCGGGUUAUUGUCACCAGAA

**Table 2:** Specific evaluation strains and results. “+” stands for target strain, The test result is positive. “−”stands for non-target strain, The test result is negative.

Serial Number	Species	Detection Results
1	*Shigella flexneri*	+
2	*Escherichia coli* O157:H7	−
3	*Enteroinvasive Escherichia coli*	−
4	*Enterotoxigenic Escherichia coli*	−
5	*Escherichia coli* O127:K63	−
6	*Escherichia coli* EPEC O86:K61	−
7	*Staphylococcus aureus*	−
8	*Cronobacter sakazakii*	−
9	*Listeria monocytogenes*	−
10	*Vibrio alginolyticus*	−
11	*Pseudomonas aeruginosa*	−
12	*Pseudomonas aeruginosa*	−
13	*Vibrio parahaemolyticus*	−
14	*Vibrio vulnificus*	−
15	*Vibrio alginolyticus*	−

## Data Availability

The original contributions presented in the study are included in the article/Supplementary Material, further inquiries can be directed to the corresponding author.

## Supplementary Materials

The following supporting information can be downloaded at: https://www.mdpi.com/article/10.3390/foods13193200/s1, Figure S1: Construction of the RPA–CRISPR/Cas12a detection platform. Exploration of the necessary components of the detection system. 1: Positive control, 2: RPA product (−), 3: Cas12a (−), 4: crRNA (−), and 5: fluorescent probe (−); Figure S2: Optimization of the RPA–CRISPR/Cas12a detection platform. (A) Optimization results of the ratio of Cas12a to crRNA. (B) Optimization results of the amount of RPA amplification product. (C) Optimization results of the amount of fluorescent probe; Figure S3: Evaluation of the sensitivity of qPCR; Figure S4: Linear relationship between the fluorescence intensity and the concentration of *Shigella flexneri* over the range of 8.3 × 10^6^ to 8.3 × 10^2^ copies/µL; Figure S5: Clinical sample detection by RPA–CRISPR/Cas12a–LFA; Table S1: A comparative analysis of existing pathogen detection methods based on CRISPR/Cas12a; Table S2: Bacterial strains used in this study. Table S3: Comparison of the sensitivity of real-time PCR and RPA–CRISPR/Cas12a–LFA for detecting *Shigella flexneri*. References [31,32,33,34,35] are cited in Supplementary Materials.
