# Supplementary material for: An RPA-Based CRISPR/Cas12a Assay in Combination with a Lateral Flow Assay for the Rapid Detection of Shigella flexneri in Food Samples
Source: Foods. 2024 Oct 9;13(19):3200. doi: 10.3390/foods13193200 (PMC11475897; doi:10.3390/foods13193200)
Supplement: Supplementary file 1 [file foods-13-03200-s001.zip › foods-3132319-supplementary.pdf]

An RPA-Based CRISPR/Cas12a Assay in Combination with a Lateral Flow Assay for the Rapid Detection of *Shigella flexneri* in Food Samples

Jieru Xu [1], Tianxin Zhang [1], Xinrui Lv [1], Lei Shi [1,2], Weibin Bai [1] and Lei Ye [1,*]

[1] Institute of Food Safety and Nutrition, Jinan University, Guangzhou 510632, China;
15713397628@163.com (J.X.); 15812492251@163.com (T.Z.);
lxrui_1995@163.com (X.L.);
shilei@jnu.edu.cn (L.S.); baiweibin@jnu.edu.cn (W.B.)
[2] Shandong Yuwang Ecological Food Industry Co., Ltd., Yucheng 251200, China
* Correspondence: yelei@jnu.edu.cn; Tel.: +86-20-85220217

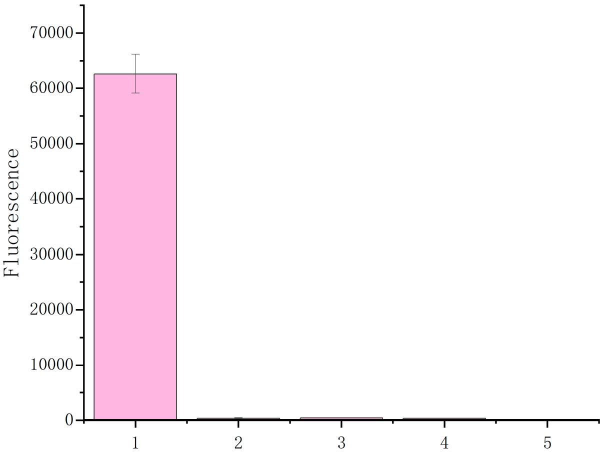

Figure S1. Construction of the RPA-CRISPR/Cas12a detection platform. Exploration of the necessary components of the detection system. 1: Positive control, 2: RPA product (-), 3: Cas12a (-), 4: crRNA (-), and 5: fluorescent probe (-).

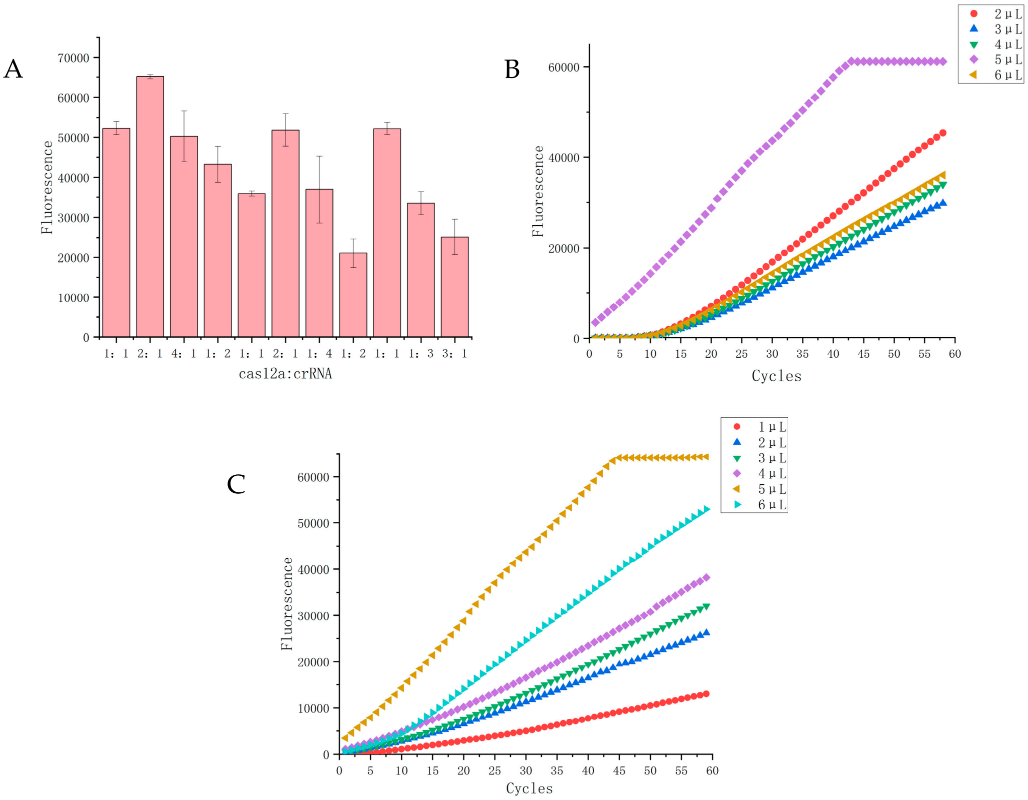

Figure S2. Optimization of the RPA-CRISPR/Cas12a detection platform. (A)Optimization results of the ratio of Cas12a to crRNA. (B)Optimization results of the amount of RPA amplification product. (C)Optimization results of the amount of fluorescent probe.

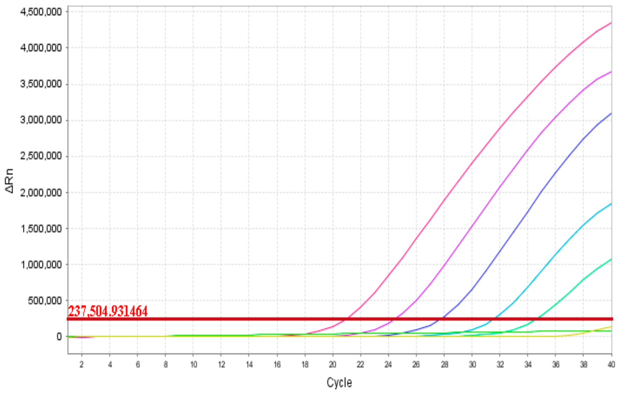

Figure S3. Evaluation of the sensitivity of qPCR.

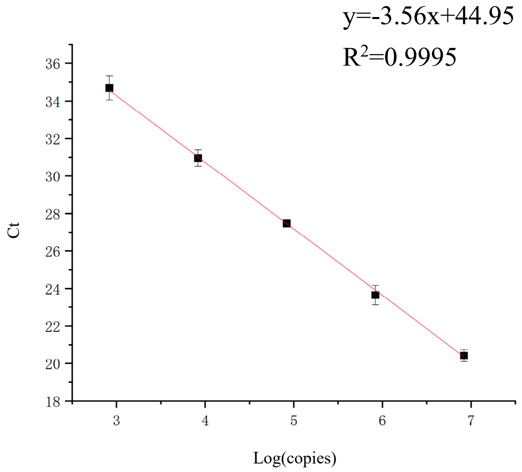

Figure S4. linear relationship between the fluorescence intensity and the concentration Shigella flexneri over the range of 8.3×10^6 to 8.3×10^2 copies/μL.

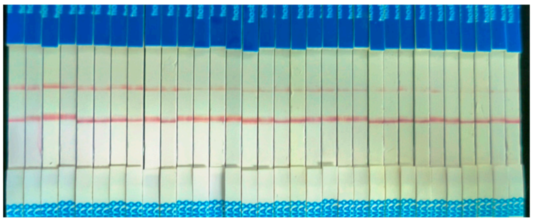

Figure S5. Clinical sample detection by RPA-CRISPR/Cas12a-LFA.

Table S1. A comparative analysis of existing pathogen detection methods based on CRISPR/Cas12a.

System name	Amplification	Sensitivity	References
RPA-CRISPR/Cas12a-LFS	E. coli O157:H7	2.5×10^2 CFU/mL	[4]
RPA-CRISPR/Cas12a-LFS	V.parahaemolyticus	1.4×10^2 CFU/mL	[5]
RPA-CRISPR/Cas12a-FT	H.pylori	50 copies	[2]
RPA-CRISPR/Cas12a	Salmonella	10^2 CFU/mL	[3]

RPA-CRISPR/Cas12a	S. aureus	1-10 copies/μL	[1]

Table S2. Bacterial strains used in this study.

No.	Bacteria strains	Strain ID	Source of isolation	Location
1	*Shigella flexneri*	ATCC12022	Reference strain	/
2	*Shigella flexneri*	SF001	Salads	Supermarkert
3	*Shigella flexneri*	SF002	Salads	Supermarkert
4	*Shigella flexneri*	SF003	Salads	Supermarkert
5	*Shigella flexneri*	SF004	Salads	Supermarkert
6	*Shigella flexneri*	SF005	Salads	Supermarkert
7	*Shigella flexneri*	SF006	Salads	Supermarkert
8	*Shigella flexneri*	SF007	Salads	Supermarkert
9	*Shigella flexneri*	SF008	Salads	Supermarkert
10	*Shigella flexneri*	SF009	Salads	Supermarkert
11	*Shigella flexneri*	SF010	Salads	Supermarkert
12	*Shigella flexneri*	SF011	Salads	Supermarkert
13	*Shigella flexneri*	SF012	Salads	Supermarkert
14	*Shigella flexneri*	SF013	salads	Supermarkert
15	*Shigella flexneri*	SF014	Salads	Supermarkert
16	*Shigella flexneri*	SF015	Salads	Supermarkert
17	*Shigella flexneri*	SF016	Salads	Supermarkert
18	*Shigella flexneri*	SF017	Salads	Supermarkert
19	*Shigella flexneri*	SF018	Salads	Supermarkert
20	*Shigella flexneri*	SF019	Salads	Supermarkert
21	*Shigella flexneri*	SF020	Salads	Supermarkert
22	*Shigella flexneri*	SF021	Salads	Supermarkert
23	*Shigella flexneri*	SF022	Salads	Supermarkert
24	*Shigella flexneri*	SF023	salads	Supermarkert
25	*Shigella flexneri*	SF024	Bananas	Supermarkert
26	*Shigella flexneri*	SF025	Bananas	Supermarkert

27	*Shigella flexneri*	SF026	Bananas	Supermarkert
28	*Shigella flexneri*	SF027	Bananas	Supermarkert
29	*Shigella flexneri*	SF028	Bananas	Supermarkert
30	*Shigella flexneri*	SF029	Bananas	Supermarkert
31	*Shigella flexneri*	SF030	Bananas	Supermarkert
32	*Shigella flexneri*	SF031	Milk	Supermarkert
33	*Shigella flexneri*	SF032	Milk	Supermarkert

Table S3. Comparison of the sensitivity of real-time PCR and RPA-CRISPR/Cas12a-LFA for detecting Shigella flexneri.

Number	Real-time PCR	RPA-CRISPR/Cas12a-LFA
SF001	33.233	+
SF002	33.920	+
SF003	28.454	+
SF004	26.903	+
SF005	34.662	+
SF006	30.404	+
SF007	33.662	+
SF008	31.789	+
SF009	36.197	+
SF010	34.899	+
SF011	33.233	+
SF012	16.790	+
SF013	18.602	+
SF014	18.891	+
SF015	36.197	+
SF016	30.759	+
SF017	33.100	+
SF018	19.471	+
SF019	20.763	+
SF020	20.583	+
SF021	21.008	+
SF022	34.828	+
SF023	31.499	+
SF024	30.759	+

SF025	22.150	+
SF026	23.127	+
SF027	23.171	+
SF028	23.895	+
SF029	32.055	+
SF030	32.828	+
SF031	25.653	+
SF032	26.555	+

References

1. Lin, L.; Zha, G.; Wei, H.; Zheng, Y.; Yang, P.; Liu, Y.; Liu, M.; Wang, Z.; Zou, X.; Zhu, H.; et al. Rapid detection of Staphylococcus aureus in food safety using an RPA-CRISPR-Cas12a assay. *Food Control* **2023**, *145*, 109505.
2. Liu, H.; Wang, J.; Hu, X.; Tang, X.; Zhang, C. A rapid and high-throughput Helicobacter pylori RPA-CRISPR/Cas12a-based nucleic acid detection system. *Clinica Chimica Acta* **2023**, *540*, 117201.
3. Liu, L.; Zhao, G.; Li, X.; Xu, Z.; Lei, H.; Shen, X. Development of rapid and easy detection of Salmonella in food matrics using RPA-CRISPR/Cas12a method. *LWT Food Sci. Technol.* **2022**, *162*, 113443.
4. Luo, J.W.; Xu, D.; Wang, J.; Liu, H.; Li, Y.; Zhang, Y.; Zeng, H.; Deng, B.; Liu, X. A Dual-mode platform for the rapid detection of O157:H7 based on CRISPR/Cas12a and RPA. *Anal. Bioanal. Chem.* **2024**, *416*, 3509–3518.
5. Wang, J.B.; Xu, D.; Liu, H.; Liu, J.; Zhu, L.; Zeng, H.; Wu, W. A visual, rapid, and sensitive detection platform for Vibrio parahaemolyticus based on RPA-CRISPR/Cas12a and an immunochromatographic test strip. *Food Qual. Saf.* **2024**, *8*, fyae008.
